# Current Status in Testing for Nonalcoholic Fatty Liver Disease (NAFLD) and Nonalcoholic Steatohepatitis (NASH)

**DOI:** 10.3390/cells8080845

**Published:** 2019-08-07

**Authors:** Hannah K. Drescher, Sabine Weiskirchen, Ralf Weiskirchen

**Affiliations:** 1 Gastrointestinal Unit and Liver Center, Massachusetts General Hospital, Harvard Medical School, Boston, MA 02114, USA; 2 Institute of Molecular Pathobiochemistry, Experimental Gene Therapy and Clinical Chemistry (IFMPEGKC), RWTH University Hospital, 52074 Aachen, Germany

**Keywords:** nonalcoholic steatohepatitis, fibrosis, grading, staging, imaging, algorithms, scores, biomarkers

## Abstract

Nonalcoholic fatty liver disease (NAFLD) is the most common chronic liver disease in Western countries with almost 25% affected adults worldwide. The growing public health burden is getting evident when considering that NAFLD-related liver transplantations are predicted to almost double within the next 20 years. Typically, hepatic alterations start with simple steatosis, which easily progresses to more advanced stages such as nonalcoholic steatohepatitis (NASH), fibrosis and cirrhosis. This course of disease finally leads to end-stage liver disease such as hepatocellular carcinoma, which is associated with increased morbidity and mortality. Although clinical trials show promising results, there is actually no pharmacological agent approved to treat NASH. Another important problem associated with NASH is that presently the liver biopsy is still the gold standard in diagnosis and for disease staging and grading. Because of its invasiveness, this technique is not well accepted by patients and the method is prone to sampling error. Therefore, an urgent need exists to find reliable, accurate and noninvasive biomarkers discriminating between different disease stages or to develop innovative imaging techniques to quantify steatosis.

## 1. Introduction

**Figure 1:**
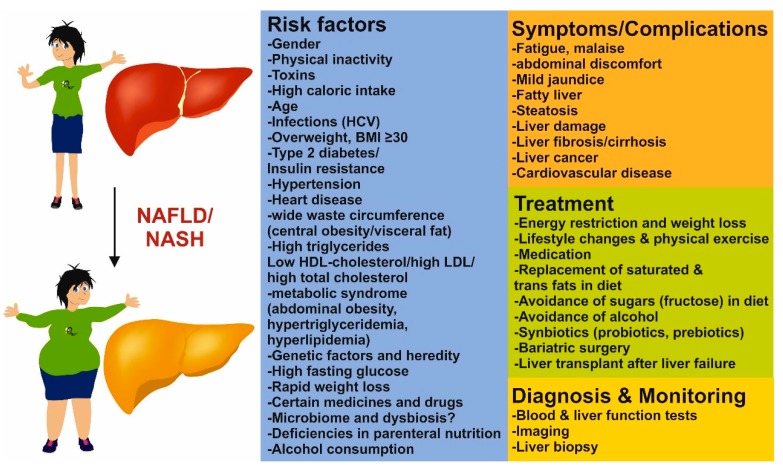
Risk factors, symptoms, diagnosis, and treatment of nonalcoholic liver disease. Predisposition and disease progression in NAFLD/NASH is influenced by comorbidities [6], genetic determinants [7], and environmental factors including drugs and toxins [8]. The range of resulting symptoms and complication of NAFLD/NASH can vary from very mild to life-threating. Diagnosis and monitoring of disease can be done by blood tests [9], liver function tests [10] and imaging [11]. However, a liver biopsy should be performed based on an individualized decision and is still the gold standard in scoring and grading of steatosis, inflammation, and fibrosis [12]. Although an ultimate therapy is still missing, beneficial effects on NAFLD/NASH progression are energy restriction, lifestyle changes, improved diets, and elevated physical activity [13]. In addition, in biopsy-proven NASH and fibrosis, medication with antiglycemic drugs, insulin sensitizers, synbiotics, or compounds interfering with fat metabolism or preventing oxidative stress have favorable effects on disease outcome [13,14]. Surgical procedures, including bariatric surgery, to treat obesity and liver transplant after liver failure are extreme forms in NAFLD/NASH treatment [15].

In the 1990s, nonalcoholic steatohepatitis (NASH) was considered to be a relatively uncommon disease, only occurring in obese women with type 2 diabetes. Things changed quickly after a study came up in 1996 describing NASH the second most commonly occurring liver disease in patients after acute and chronic viral hepatitis infection [1]. Even more important for the field of NASH was a review in 1998 highlighting that fibrosis and cirrhosis are complications associated with NASH in more than 15–50% of all cases [2]. In the same article, Day and James postulated the two-hit hypothesis according to which the pathogenesis of NASH is initiated by a first hit predominantly caused by accumulating lipids in hepatocytes leading to apoptosis of these cells and excessive oxidative stress. Steatosis then sensitizes the liver to develop advanced NASH by a potential second hit driven by infiltrating immune cells, which release inflammatory mediators such as cytokines. This shift to an inflammatory milieu can finally lead to fibrotic tissue remodeling which can easily progress to end stage liver disease such as cirrhosis and the development of hepatocellular carcinoma (HCC). Today, this hypothesis is considered to be inadequate to explain the multiple and complex disease drivers to nonalcoholic fatty liver disease (NAFLD). Lately the multiple-hit hypothesis is better framing the variable circumstances implicating disease development and progression [3]. However, studies show that disease progression is not always linear and it is not fully clear who is more likely to progress to more advanced stages [4]. Considered as the hepatic manifestation of metabolic syndrome, NAFLD is in most cases associated with type 2 diabetes and dyslipidemia (Figure 1) [5].

Taking the direct association with insulin resistance and obesity into account, NAFLD is a global health burden with rising impact [16,17]. It is known that almost 25% of the global population is affected by NAFLD and/or its complications, making it the most common chronic and progressive liver disease especially in industrialized countries [18]. Models even predict a prevalence of NAFLD in adults of more than 30% of the total population with around 20% being diagnosed as NASH. Looking at these developments, calculations show that the incidence of HCC and NASH-related liver transplantation will be doubled until 2030 [19,20,21].

**Figure 2:**
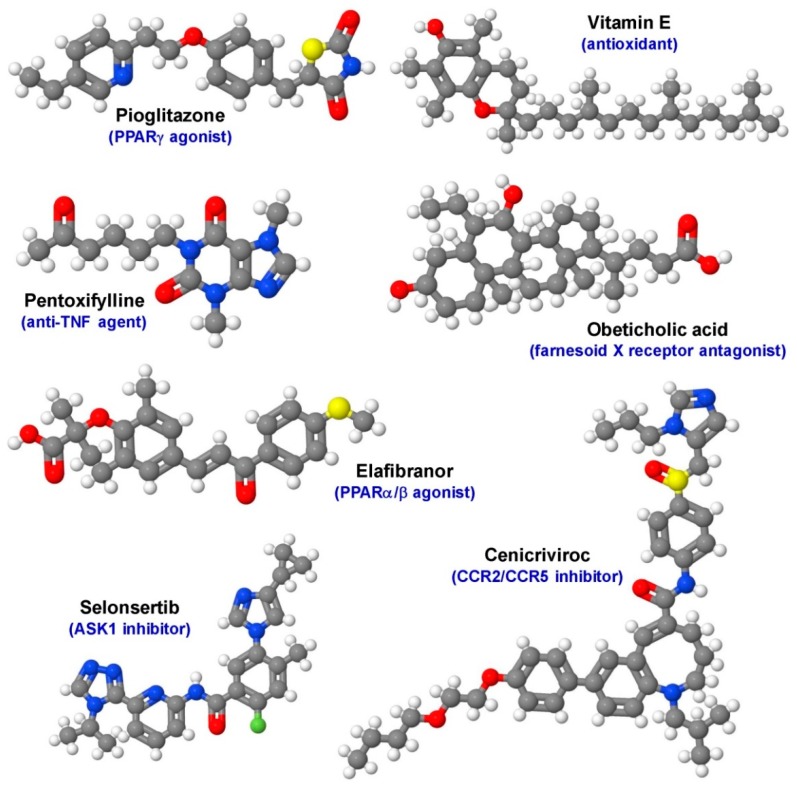
Established compounds and drug candidates evaluated for the treatment of NAFLD/NASH in clinical trial phase III. Pioglitazone (PPARγ agonist), vitamin E (antioxidant), pentoxifylline (anti-tumor-necrosis factor-α (TNF-α) agent), Obeticholic acid (farnesoid X receptor antagonist), cenicriviroc (CCR2/CCR5 inhibitor), elafibranor (PPARα/δ agonist), and selonsertib (ASK1 inhibitor) have different molecular targets. The different biological activities of the drugs point to the complexity of NAFLD/NASH, having a large variety of potential therapeutic drug targets. The depicted structures were generated with the open source molecule viewer Jmol using data deposited in the PubChem compound database with CIDs: 4829, 14985, 4740, 447715, 11285792, 9864881, and 71245288). For more details about the biological activity of each drug refer to [32].

Up-to-date weight loss and lifestyle changes are still the only possible ways to overcome NAFLD. Regrettably, very few patients successfully achieve long term weight reduction. Therefore, there is an urgent need for the development of a pharmacological treatment for patients with NASH and fibrosis. NASH and fibrosis typically develop asymptomatic till they progress to end-stage liver disease at which liver transplantation is the only cure available. At present, first guidelines consent to the use of pioglitazone, vitamin E and pentoxifylline in patients with NASH to reduce steatosis, but they still have many potential side-effects or have low efficiency [22,23,24,25]. Other agents such as obeticholic acid, cenicriviroc, elafibranor and selonsertib are actually in clinical trial phase III (Figure 2) [26]. Although these various clinical trials show promising results to target NAFLD and NASH therapeutically, there is no approved pharmacological treatment available yet [27,28,29,30,31].

On the basis of different imaging techniques and the combination of clinical factors the presence of NASH in an individual can be strongly suspected. However, the gold standard to diagnose NASH is still an invasive liver biopsy [4]. This is not only less accepted and harmful to the patient, but also often leading to late diagnoses at the point of end-stage liver disease due to the fact that NAFLD is in most cases asymptomatic prior to the transition to NASH. In addition, not all researchers agree with the view that there exists a necessity of differentiating by histology the so-called simply or benign fatty liver from NASH. This is due to the fact that NAFLD may be less benign than it is currently thought to be and that there exists a high degree of heterogeneity of NAFLD [33].

Twenty to thirty percent of all NAFLD patients progress to NASH-fibrosis. Therefore, there is an urgent need to find reliable noninvasive biomarkers and screening techniques to diagnose NAFLD and NASH and monitor patients at an earlier time point at which lifestyle changes and potential newly developed drugs can be used purposefully.

In addition, other less common conditions can cause similar clinical and histological phenotypes like NAFLD and NASH [34]. There is common sense that the primary causes for the development of NAFLD are obesity, type II diabetes, dyslipidemia, insulin resistance and some genetic disorders [34]. However, there are other less common conditions in which NAFLD is the consequence of secondary causes, including specific disorders of lipid metabolism (abetalipoproteinemia, hypobetalipoproteinemia, familial combined hyperlipidemia, glycogen storage disease, Weber–Christian syndrome, lipodystrophy), total parenteral nutrition, hepatitis C infection, severe surgical weight loss, medications (amiodarone, tamoxifen, methotrexate, corticosteroids, highly active antiretroviral therapy), starvation, Wilson’s disease, environmental toxicity, and celiac disease [34].

Some other conditions associated with metabolic syndrome that can lead to NAFLD are the obstructive sleep apnea syndrome (OSAS), polycystic ovary syndrome (PCOS), and non-obese NAFLD. OSAS is a common sleep disorder, which is associated with chronic intermittent hypoxia and increased proinflammatory cytokine production [35]. OSAS and obesity often coexist and the chronic hypoxia induces hyperglycemia, insulin resistance, and hepatic lipid peroxidation, which are hallmarks of the metabolic syndrome [35]. Similarly, PCOS is a frequent endocrine disease in women associated with a number of metabolic consequences, including obesity, dyslipidemia, insulin resistance, type 2 diabetes, and low-grade inflammation [36]. Therefore, it is not surprising that PCOS patients are prone to develop NAFLD. Interestingly, NAFLD can also occur in non-obese individuals. Although these patients have a normal body mass index (BMI), they have metabolic abnormalities similar to those characteristically associated with obesity [37]. Consequently, these normal weight or lean individuals displaying obesity-related features are called metabolically obese but normal weight (MONW) patients [37]. Although the pathways and pathophysiological mechanisms driving NAFLD and NASH in non-obese persons are not completely understood, it is speculated that major risk factors are dysfunctional fat, decreased muscle mass, genetic factors, different patterns of the gut microbiota, and epigenetic changes occurring early in life [38].

To avoid diagnostic pitfalls in the distinction of NAFLD provoked by traditional risk factors of metabolic disease and secondary causes of NAFLD, it is essential that clinicians comprehensively evaluate the patient, because both conditions vary considerably and will require different therapeutic regimens [34,39].

This review summarizes the current screening methods used to complement the overall appearance of NAFLD/NASH and provides an outlook on potential upcoming candidates to replace the need of taking liver biopsies.

## 2. Blood and Serum Tests

Liver biopsy is still the standard procedure to diagnose NASH. Besides the risk of complications during surgery, it involves a lot of bias because only a little specimen of the liver is taken, which not always represent the actual status of the entire liver [40]. This runs the risk to underestimate disease severity in many cases [41]. Finding reliable biomarkers which can be measured with less or even noninvasive techniques is therefore of urgent need. 

However, NAFLD and NASH are complex multi factorial diseases and therefore no single surrogate marker is likely to be omniscient to predict clinical outcome or benefits of a therapy. Despite the fact that all biomarkers and scores have their limitations, interest is increasing rapidly in the use of these markers to predict information about progression and outcome of the disease. Therefore, respective surrogate biomarker and scores offered by the market should be used with much care and limited to situations where it has been demonstrated robust ability in disease management. In addition, there is an urgent need to improve standardization in the usage of these operations. On the other site, it is obvious that the surrogate markers can be extremely helpful when handled correctly. This was very recently demonstrated in a study using telemedicine-based comprehensive, continuous care intervention (CCl) together with carbohydrate restriction-induced ketosis and behavior changes. The respective study showed that a NAFLD liver fat score (i.e., N-LFS) was reduced in the CCl group, whereas it was not changed in a group of patients receiving usual care [42]. This exemplarily demonstrates that surrogate markers can provide good measurement for the efficacy of a specific therapy.

Here we will summarize blood and serum biomarkers, which are already available and discuss their benefits and shortcomings in the diagnosis and management of NASH and NAFLD.

### 2.1. Steatosis

Hepatic steatosis is the key feature of NAFLD. Steatosis is diagnosed when more than 5% of hepatocytes contain fat or when the total amount of intrahepatic triglycerides is bigger than 5.5% without having any other liver disease in the patient’s history [23,24]. Today there is no specific serum marker to assess hepatic steatosis available. However, several reproducible blood biomarker panels and scores were developed to help diagnose NAFLD (Table 1).

**Figure 3:**
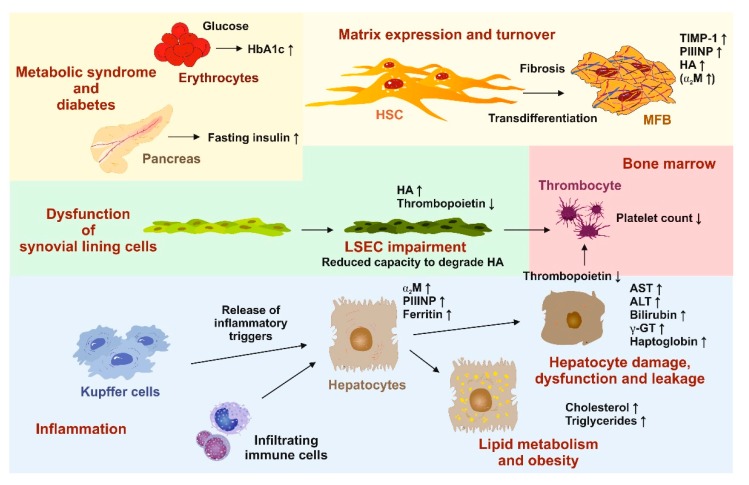
Significance of laboratory parameters in the diagnosis NAFLD and NASH. In the scheme, the biological source and alteration of individual biochemical markers during progression of NAFLD/NASH are indicated. High caloric intake and elevated quantities of fat result in hepatic steatosis, triggering steatohepatitis. The inflammatory response is triggered by infiltrating immune cells and liver-resident Kupffer cells releasing a plenitude of inflammatory triggers. As a consequence, the expression of acute phase response proteins (α_2_ macroglobulin (α_2_M) and ferritin) is increased in hepatocytes. In addition, the increase of cholesterol and triglycerides provokes cellular fat accumulation, damage, and cellular leakage of hepatocytes. This is indicated by elevated quantities of aspartate aminotransferase (AST), alanine aminotransferase (ALT), bilirubin, and γ-glutamyltransferase (γ-GT). Subsequently, the overall capacity of these cells to synthesize typical liver proteins (haptoglobuin, thrombopoietin) decreases. Lower quantities of thrombopoietin results in reduced formation of platelets within the bones. Dysfunction of synovial lining cells (reduced capacity to degrade hyaluronic acid (HA)) and ongoing fibrogenesis lead to elevated levels of HA. In addition, the transdifferentiation of hepatic stellate cells (HSC) to myofibroblast (MFB) is associated with the occurrence of typical biomarkers (tissue inhibitor of matrix metalloproteinase-1 (TIMP-1), amino-terminal propeptide of type III procollagen (PIIINP)), which correlate to extracellular matrix formation and/or turnover. The metabolic syndrome associated with NAFLD/NASH results in higher quantities of fasting insulin and basal glucose triggering the non-enzymatic formation of glycated hemoglobin (HbA1c). All these parameters are diagnostically relevant in the diagnosis or scoring of NAFLD/NASH and are the basis of various blood biomarker panels to identify inflammatory liver disease.

Most of these multiparametric panels include biochemical markers indicating liver damage or dysfunction (AST, ALT, bilirubin, γ-GT, platelet count, haptoglobin), lipid metabolism disorders (cholesterol, triglycerides), diabetes (HbA1c, fasting insulin level), inflammation (α_2_M, ferritin), or provide information about matrix expression and turnover (TIMP-1, PIIINP, HA) (Figure 3).

With an AUROC (area under the receiver-operating characteristic curve) accuracy value of 0.87, the NAFLD ridge score is currently one of the most efficient panel based on laboratory parameters. The NAFLD ridge score was developed as a machine learning algorithm facilitating registry research. It includes serum levels of alanine aminotransferase (ALT), high-density lipoprotein (HDL) cholesterol, triglycerides, hemoglobin A1c (HbA1c), leukocyte count, and the presence of hypertension [43,44]. With proton magnetic resonance spectroscopy (H-MRS) as reference, the NAFLD ridge score has a negative predictive value of 96%. However, this score is good to detect NAFLD but yet limited to the research setting and does not give the opportunity to distinguish between different steatosis grades or to assess changes during the development of steatosis over time.

A quantitative and by this more sensitive score to be calculated is the NAFLD Liver Fat Score (NLFS). This score includes the measurement of the liver fat content as determined by H-MRS, the presence or absence of the metabolic syndrome together with type 2 diabetes mellitus, aspartate aminotransferase (AST) levels, the AST:ALT ratio, and the fasting insulin serum level. With a sensitivity of 86% and a specificity of 71% the NLFS defines a liver fat content of more than 5.56% [45]. A recent study from Ruiz-Tovar and colleagues tested the accuracy of the NLFS in patients one year after bariatric surgery and considered it to be the most accurate biochemical score to assess liver steatosis at the moment [61]. The Hepatic Steatosis Index (HIS) also considers the AST/ALT ratio, BMI, diabetes and sex and has a sensitivity of 66% and a specificity of 69% [46].

The fatty liver index (FLI) includes BMI, waist circumference and serum levels of triglycerides and the γ-glutamyltransferase (γ-GT). It could be shown that the FLI significantly correlates with insulin resistance [47,48]. The major drawback when rating HIS and FLI is that ultrasonography is used as the reference standard to diagnose fatty liver. This technique is in general dependent on the operator and thereby to some extent biased and insensitive if only mild steatosis is present. The lipid accumulation product index (LAP) first established by Bedogni et al. takes into account sex, serum triglyceride levels and weight circumference to evaluate lipid overaccumulation [49].

A comparison of the accuracy in predicting NAFLD in a cross-sectional NAFLD cohort showed that NLFS is the best score to reliably predict NAFLD with an AUC of 0.771 [62]. Although the presented scores are capable to indicate the presence of hepatic steatosis, there are several limitations given. To be critically considered are the facts that using these indices it is not possible to distinguish between different steatosis grades and detect and trace changes over time is not possible.

### 2.2. Steatohepatitis

The transition from simple hepatic steatosis to NASH is the most crucial step during the development of severe liver disease with poor prognosis and the higher risk to get fibrosis and progress to end-stage liver disease. Thus, the assessment of NASH and the possibility to distinguish between the dynamic changes from NAFLD to NASH are ongoing challenges. Precise diagnosis still depends on liver biopsy with huge variability between pathologists. For that reason, Bedossa et al. developed the Fatty Liver Inhibition of Progression (FLIP) algorithm, which requires pathologists to follow generalized criteria for scoring. The FLIP algorithm considers histologically steatosis, disease activity and fibrosis scores [50]. Very recently, Canbay and colleagues established a novel machine learning approach to assess the severity of NAFLD and distinguish between NAFLD and NASH. In this study NAFLD was defined as the NAFLD activity score (NAS) ≤ 4 and NASH as NAS ≥ 4. With the help of an ensemble feature selection approach (EFS) they identified age, HbA1c, γ-GT, adiponectin and the apoptosis marker M30 to be the biomarkers highly associated with the prediction of NAFLD. The developed CHeK score, which is available at http://CHek.heiderlab.de is not only able to detect NASH, but also to monitor the development from NAFLD to NASH and can be used to screen patients in a long-term follow up during disease progression or therapy [51].

Besides histological scoring, the development from NAFLD to NASH involves a variety of different molecular, cellular and hormonal changes. Numerous blood biomarker and panels were investigated and developed trying to detect and reflect disease severity and underlying pathways. The apoptosis marker cytokeratin 18 (CK18) is a very well-studied individual blood biomarker so far. NASH patients show a significant increase of plasma CK18 indicating hepatocyte death through apoptosis and necroptosis compared to NAFLD patients [63]. CK18 is the main intermediate filament protein in hepatocytes and is released upon the initiation of cell death [64]. While the whole length CK18 is predominantly released upon hepatic necrosis, caspase cleaved CK18 (M30) is mainly produced by apoptotic cells [65]. Although CK18 is considered to be one of the most promising biomarkers, several studies showed that the sensitivity to predict NASH is 66%, while the specificity is 82% [66,67]. In addition, the ability of M30 to predict NASH and distinguish between NAFLD and NASH was calculated as 0.82 [68]. To increase the reliability of CK18 as a noninvasive biomarker for NASH a study shows that the combination with serum levels of the apoptosis-mediating surface antigen FAS (sFAS) further increases the accuracy [69]. However, the optimal cut-off serum concentrations still vary between different studies and require further investigation.

NASH is predominantly characterized by pathological alterations in glucose and lipid metabolism. These alterations include modifications in adipokines (such as leptin, adiponectin and resistin) and liver-derived lipid hormones like the fibroblast growth factor 21 (FGF21), which is secreted upon peroxisome proliferator-activated receptor-α (PPARα) activation [70,71]. FGF21 was found to be significantly elevated in patients with mild to moderate hepatic steatosis. Serum levels were directly linked to increased intrahepatic triglyceride accumulation and liver damage [72,73]. However, FGF21 is known to also increase in sepsis and systemic inflammation [74]. Further, adipokines were shown also to reflect visceral adiposity leading to a moderate specificity value of 62% with a specificity of 78% [68]. Further studies even show a drop of FGF21 levels with increasing liver inflammation [75].

The most evident difference between simple steatosis and advanced steatohepatitis is the absence of an inflammatory infiltrate. As a hallmark of NASH, a variety of inflammatory markers are elevated in patients with NASH, while disease is progressing. Increasing serum levels of C-reactive protein (CRP), tumor necrosis factor-α (TNF-α) and several interleukins such as, IL-6 and IL-8 were proposed as clinical markers. Although, they all correlate with the observed inflammatory status in NASH, none of them reached statistically significant values adjusted by the FDR on univariable analysis to be approved as a diagnostic marker yet because of their insensitivity to NASH specific inflammatory changes.

Recently, the transcription factor forkhead box protein A (FOXA1), also known as hepatocyte nuclear factor 3-α, was described as a potential new biomarker as it is involved in mediating homeostasis and metabolism by targeting genes in liver, adipose tissue and pancreas [76]. Moya et al. could show that FOXA1 acts anti-steatotic by lowering fatty acid uptake and is suppressed in patients with NAFLD and insulin resistance [77]. Therefore, the authors proposed this protein as sensitive noninvasive biomarker of liver fat accumulation, mitochondrial membrane potential and the production of reactive oxygen species (ROS). The limitation coming along with using a transcription factor as biomarker is, that FOXA1 is not secreted into the serum.

Oxidative stress, which is indicated by excessive ROS production, is one of the most important mechanisms underlying the disease pathogenesis of NASH finally leading to lipid oxidation and inflammation [78]. Based on changes in lipid catabolism and de novo lipogenesis the oxNASH score was calculated including the linoleic acid:13-hydroxyoctadecadienoic acid (13-HODA) ratio together with the patient characteristics age, BMI and AST level. This score reached diagnostic accuracy with an AUROC 0.74–0.83 [79]. Because mass spectroscopy is needed for the measurement of the described parameters, the oxNASH score is not commonly used today. In line with biomarkers targeting products, which are secreted due to an altered lipid metabolism, insulin-like growth factor binding protein 1 (IGFBP-1) was recently suggested as a potential serum marker for NAFLD and NAFLD-related fibrosis. It is exclusively upregulated in the liver in response to hepatic inflammation and oxidative stress and regulated by insulin [80]. On this basis, Regué et al. could show that the global deletion of the insulin-like growth factor 2 mRNA-binding protein 2 (IGF2BP2 or IGF2 mRNA-binding protein 2, IMP-2) lead to a resistance to obesity and fatty liver in mice treated with a high fat diet (HFD) due to reduced adiposity [81]. A limitation of those markers is that elevations might be not exclusively related to NAFLD-induced conditions, but also the metabolic syndrome and insulin resistance in general. Anyhow this is an interesting starting point for future investigations also in regard to therapeutic interventions and the understanding of the mechanisms that lead to steatosis.

The expression of ferritin is generally known to be increased in patients with NAFLD and metabolic syndrome. It was further shown to be independently associated with increased steatosis grades, NASH and NASH fibrosis with an AUROC of 0.62 [82,83]. This accuracy can be increased to an AUROC of 0.81 when including AST, BMI, type 2 diabetes, presence or absence of hypertension and platelet count to ferritin levels [84]. The broad and long-lasting search for novel biomarkers to diagnose NASH, which are modestly accurate, show the multiple factors involved in NAFLD and the complexity of disease mechanisms. To date the combination of several biomarkers drastically increases diagnostic preciseness. Especially for NASH, panels like the Nash Test (NT) include baseline patient characteristics such as age, gender, height, weight and serum levels of triglycerides, cholesterol, transaminases, total bilirubin, α_2_-macroglobulin, haptoglobin, apolipoprotein A1, γ-GT [85].

Overall, most of the actual biomarkers and panels need further validation on cohorts with patients, including several different ethnicities and various starting points and outcomes. Up to now most validation studies work with patients undergoing bariatric surgery. Also choosing the best cut-off value for the specific serum markers is still not optimal. This points to the urgent need of basic research studies to help better understanding the underlying mechanisms and key molecules involved in the development of NAFLD and progression to NASH and end-stage liver disease.

### 2.3. Fibrosis

Studies show that the F2 stage of fibrosis is one of the most critical points in the progression from NASH and NASH fibrosis to end-stage liver disease, making it a crucial step for therapeutic intervention [86,87]. The risk of liver-specific mortality at stages F3 and F4 fibrosis is shown to increase by 50–80%. Thus, diagnosis and monitoring patients with noninvasive strategies is a major focus of actual research. Effective clinical NASH treatment is achieved when fibrosis progression is prevented and/or fibrosis is improved.

Most biomarkers do not measure fibrogenesis or fibrinolysis directly. Thus, those indirect surrogate markers show a low accuracy leading to the necessity of biomarker panels to improve their reliability on the discrimination between different fibrosis stages. The most common scores that combine several clinical parameters are the NAFLD Fibrosis Score (NFS), the Fibrosis-4 Score (FIB-4), the AST to Platelet Ratio Index (APRI) and the BARD Score, which includes BMI, AST:ALT ratio and diabetes.

The NFS includes several generally measured parameters and is well-studied in regards to its accuracy [45]. Simple online calculation of the respective score can be done free of charge at http://www.nafldscore.com/. Taking into account the AST:ALT ratio, albumin, platelet count, age, BMI and hyperglycemia, the NFS has a high predictive value, thereby avoiding the need of liver biopsy in many patients [45]. Nevertheless, there are two different cutoff level described to either exclude or diagnose advanced fibrosis. This is leading to the problem that patients who end up with scores in between the two cutoff levels are not classified properly.

The FIB-4 index described in 2010 by McPherson et al. has an accuracy of AUROC 0.86 for advanced fibrosis and relies on the AST, ALT, platelet count and age [53]. With a high negative predictive value of more than 90% and a positive predictive value of 82% the FIB-4 index is one of the reliable fibrosis scores to avoid liver fibrosis for diagnosis. Also, for the FIB-4 index there are two different cutoff level, i.e., a score <1.45 for moderate and >3.25 for advanced fibrosis [88]. Both, the NFS and FIB-4 scores have been shown to be capable to predict decompensation in patients with NAFLD and NASH [89,90].

Modified by the diagnosis of chronic hepatitis C is the APRI index calculating the AST/platelet ratio. Based on its simplicity to be calculated the APRI index has a comparably low accuracy with AUROC 0.788 to predict advanced fibrosis but is highly feasible as few and very common markers are used [54]. An online tool for calculating and interpretation of APRI index results can be found at: https://www.hepatitisc.uw.edu/go/evaluation-staging-monitoring/evaluation-staging/calculating-apri.

The BARD score, including the presence of type II diabetes, BMI and the AST:ALT ratio, comes with an AUROC of 0.81 to detect F3 fibrosis. Developed by Harrison et al. in 2008, this score has a high negative predictive value of 96% whereas the positive predictive value is modest [55].

Very recently the MACK-3 was proposed as a marker for fibrotic NASH. MACK-3 includes the HOMA insulin resistance, AST and CK18 serum level. With an AUROC of 0.80 and a negative predictive value of 100% for fibrotic NASH and 74% for active NASH MACK-3 seems to be a promising score for future investigation and validation [91].

Taken together, the scores that are actually available still have only moderate sensitivity and further investigation on noninvasive markers is urgently needed. Although all scores have comparable high negative predictive values and use common parameters measured during the general blood work so that they are easy to calculate and are definitely useful to screen patients, which are at risk to develop NAFLD related fibrosis and end-stage liver disease.

The measurement of specific fibrosis biomarkers in serum such as hyaluronic acid [92], procollagen III amino-terminal peptide (PIIINP) type IV collagen [93], TIMP-1 (tissue inhibitor of metalloproteinase 1) [94] or laminin [95] did not reach clinical routine, although they correlate with NASH and fibrosis with AUROC ranging from 0.87 (for hyaluronic acid) to 0.97 (for TIMP-1) [96]. The reason for that is most likely that measurement is cost-intensive and technically complex.

Further developments in the field combine different serum parameters in complex algorithms such as the Enhanced Liver Fibrosis panel (ELF) [56], FibroTest/FibroSURE/ActiTest [58], FibroMeter NAFLD index [59,60], Hepascore [57], and many others show very promising results to diagnose and distinguish patients with F0-F2 fibrosis from those with F3-F4 fibrosis. Those algorithms have to be validated in the clinics and have to be further developed and simplified to be able to make them widely applicable.

For the validation of a new diagnostic test method, the STARD checklist (Standards of Reporting of Diagnostic Accuracy Studies) was established and published by 13 journals in 2003 and modified to also meet the criteria needed for the evaluation of liver fibrosis in 2015. This Liver-FibroSTARD checklist should help to reach consent on the requirements for new noninvasive fibrosis markers [97,98].

However, it is obvious that the prediction of NASH severity by a noninvasive fibrosis marker, score, a diagnostic test, or an algorithm incorporating a panel of biomarkers is not necessarily capable of making a comprehensive statement of the disease outcome. Confounding factors, comorbidities, or simple blood parameters can significantly impact the progression or overall outcome of NASH. This was recently documented in a cross-sectional study in which 100 obese patients suffering from hepatic steatosis were analyzed for the occurrence of atherosclerosis [99]. Interestingly, the authors found that a lowered copper bioavailability is linked to atherosclerosis, which is the main complication of NAFLD. In line, reduced hepatic copper concentrations were found in human NAFLD patients and associated with higher degrees of hepatic steatosis in rats fed with low dietary copper [100].

## 3. Imaging

Although histological evaluation of liver biopsies and calculation of values that are based on the determination of serum markers and measurement of body features are extremely helpful to get information on inflammation and fibrosis, these methods have also some limitation. In particular, liver biopsy is invasive, relative costly and associated with relevant biopsy-related complications including bleeding, pain and infections. Moreover, it has been known for decades that the procedure has a high inter-observer variability and may not be representative for the whole organ [101]. In addition, it is impossible to apply liver biopsy to monitor changes in fibrosis stages because this would require multiple repetition of the procedure. Similarly, single or combination of biomarker measurements, which meet all the diagnostic criteria required for widespread, cost-effective, and reliable use are not available. A major pitfall in all these measurements is that the evaluation of any new biomarker is hindered by the lack of reference tests and the inter-technique analytical variability and performance of individual parameters. Therefore, individual biomarkers or multiparametic panels of biomarkers have reached only limited clinical application. Imaging techniques, providing direct information about the health status of the liver, have therefore emerged as attractive alternatives to assess steatosis, steatohepatitis, fibrosis, cirrhosis or hepatic cancer. In the following we will briefly summarize of imaging-based methods for diagnosing NASH and NAFLD.

### 3.1. Ultrasound

Historically, the first report on using ultrasonography as a diagnostic tool in hepatic steatosis and steatohepatitis was published in 1981, in which patients suffering from alcohol-related disease were analyzed for parenchymal alterations, fatty infiltration, dilatation of hepatic veins and ascites [102]. Later studies have shown that the sensitivity of this radiologic modality is somewhat limited when the content of hepatic steatosis is below a certain threshold [103]. However, by use of this imaging technique, it was demonstrated in a prospective study investigating a cohort of 400 patients that the overall prevalence of NAFLD and NASH is significantly higher than originally estimated [104]. Based on these findings, the method is a significant diagnostic add-on for screening of patients at risk for NAFLD and NASH, in particular when liver enzymes are elevated. In line with this assumption, a very recent descriptive, cross-sectional study categorizing 109 patients into different grades of NAFLD by ultrasonography showed that ultrasound is still an important, cheap, and easy-to-use imaging tool for the diagnosis and grading of fatty liver diseases [105]. In addition, the authors could demonstrate that ultrasound is ideally suited to manage patients with fatty liver in follow-ups, because of its significant association with deranged lipid profiles and the lack of any side effects.

Contrast-enhanced ultrasound (CEUS) allowing to monitor not only qualitatively, but also quantitative analysis of lesion microcirculation has helped to establish diagnostic procedures for detection of focal and diffuse liver pathologies and to assess a differential diagnosis between benign and malignant liver lesions [106].

### 3.2. Ultrasound-Based Elastography Techniques

In 2005, it was demonstrated that the propagation and velocity of low-frequency pulsed shear waves in real time in biological tissue is directly correlated to the amount of extracellular matrix [107]. Respective methods relying on the velocity of shear waves, introduced as “transient elastography”, “vibration-controlled transient elastography (VCTE)” or “real-time shear wave elastography (SWE)” are particularly suited to detect liver cirrhosis by high stiffness in which it has a diagnostic specificity of 99% [108]. It is noteworthy, that a VCTE device such as the FibroScan introduced by Echosens SA can be performed as a point-of-care (POCT) test at the place of patient care enabling test results to be immediately shared instantly with care providers or patients. Unfortunately, the device has potential technical limitations in patients suffering from ascites, high quantities of chest wall fat, and in individuals who are morbidly obese [109].

In acoustic radiation force impulse elastography (ARFI) measuring beam passes over a standardized region of interest of the liver, obesity as defined by a BMI of larger 30 kg/m^2^ or ascites are not considered obstacles [110]. However, ARFI imaging was found to have a poor diagnostic performance in patients with BMI larger than 35 kg/m^2^ [111]. Other ultrasound real-time imaging modalities for soft tissue elasticity mapping are SWE or supersonic shear imaging (SSI) [104]. In these measurements, the propagation of the waves is stored in small video clips from which the elasticity of the analyzed tissue can be mapped quantitatively [112]. Although, these methods have good to excellent performance for the noninvasive staging of fibrosis in hepatitis B infected patients, data on other liver disease cohorts are still needs to be established [113].

However, in some disease conditions, the combination of elastography techniques with special NAFLD scores composed of simple measures of patient characteristics and clinical chemistry parameters increase the overall accuracy in assessment of clinically significant liver fibrosis in NAFLD [114]. The combination of various methods is therefore potentially relevant to better identify patients in which a liver biopsy is appropriately indicated.

### 3.3. Controlled Attenuation Parameter

The controlled attenuation parameter (CAP) was developed specifically for the FibroScan device to allow detection of hepatic steatosis in patients with about 10% of fatty hepatocyte degeneration without being influenced by liver fibrosis or cirrhosis. This threshold is clinically highly relevant because the diagnosis of steatosis is generally made when hepatic lipid content exceeds 5–10% by weight [115]. A significant correlation of the CAP signal and steatosis was first demonstrated in 2010 [116]. This study showed that CAP can efficiently separate grades of steatosis with AUROC values of 0.91 and 0.95 for the detection of more than 10% and 33% of steatosis. However, although these characteristics are diagnostically promising, it should be critically mentioned that CAP increases after a meal across all stages of fibrosis, potentially leading to misclassification of patients when the operator does not adhere to preanalytical necessities [117].

### 3.4. Magnetic Resonance Imaging in NASH and NAFLD

Magnetic resonance imaging (MRI) provides another possibility to quantify hepatic fat content with high spatial resolution. Like the other imaging techniques mentioned before, MRI scans do not require or emit ionizing radiation. However, the generation of meaningful MRI images in high resolution requires long imaging times that can be shortened by intravenous administration of gadolinium(III)-based contrast agents [11]. Advanced MRI techniques such as MRI proton density fat fraction (MRI-PDFF) were developed to specifically detect the presence of hepatic steatosis and to assess liver fat over the entire liver [118]. In this analysis, the PDFF is given as an absolute percentage ranging from 0% to 100% and defined as the ratio of density of mobile protons from fat (i.e., triglycerides) and the total density of protons from mobile triglycerides and mobile water [119]. Based on its robustness, practicability, reproducibility PDFF was proposed as the best-suited quantitative MR-based quantitative MR-based biomarker of tissue fat concentration for large-scale research endeavors and widespread clinical implementation [120]. Several independent studies analyzing NAFLD patients showed that MRP-PDFF performed better than CAP for diagnosing all stages of steatosis and had an overall better diagnostic accuracy [121,122,123].

### 3.5. Magnetic Resonance Elastography

A meta-analysis including retrospective studies showed that magnetic resonance elastography (MRE) is particularly useful to determine liver stiffness and has high accuracy for the diagnosis of significant or advanced fibrosis and cirrhosis [124]. Therefore, this imaging modality may be highly suitable to detect progression and treatment response in patients with chronic liver disease. This imaging technique is complementary to ultrasound-based elastography techniques and is highly accurate in diagnosing advances fibrosis in patients suffering from NAFLD [125]. This was documented in a prospective study in which the accuracy of 3D-MRE and 2D-MRE was compared in a cohort of 100 consecutive patients with biopsy-proven NAFLD [126]. This study further revealed that 3D-MRE is significantly more accurate than 2D-MRE for diagnosis of advanced fibrosis in NAFLD patients.

MRE was also found to be more accurate than ultrasound-based transient elastography in a cross-sectional study of more than 100 NAFLD patients in which fibrosis were detected with an AUROC of 0.82 (95% confidence interval, 0.74–0.91) [122]. Also, for classification of steatosis and necroinflammtory activity, MRE showed higher diagnostic performance than transient elastography in patients with NAFLD [121,127].

## 4. Genetic Tests

**Figure 4:**
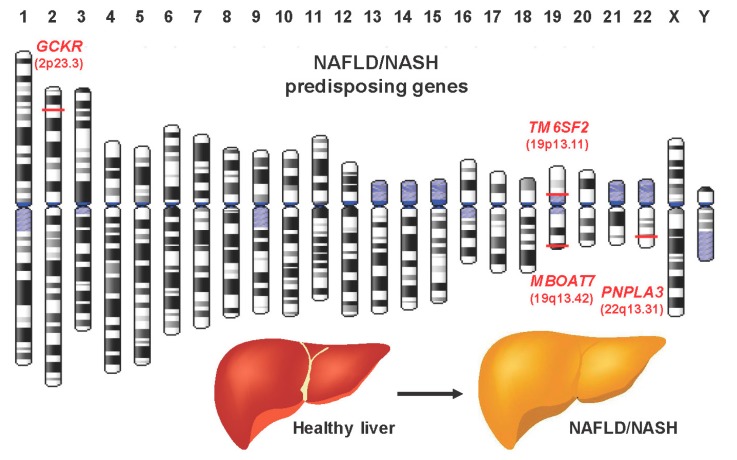
Gene alterations in NASH. Gene alterations/modifications associated with the pathogenesis of NASH affect the *GCKR*, *TM6SF2*, *PNPLA3*, and *MBOAT7* genes. The chromosomal location of respective genes is depicted in the ideogram. Gene annotations were done with the Genome Decoration Page (https://www.ncbi.nlm.nih.gov/genome/tools/gdp) and genomic coordinates deposited in the Online Mendelian Inheritance in Man (OMIM) database under accession no. 609567 (*PNPLA3*, 22q13.31), 606563 (*TM6SF2*, 19p13.11), 606048 (*MBOAT7*, 19q13.42), and 600842 (*GCKR*, 2p23.3).

Genome wide association studies increased our knowledge and understanding of genetic and genomic alterations and components during NASH development and progression leading to the identification of several potential target genes, not only for therapeutic intervention, but also for the prediction of risk patients. The most abundant alterations are genetic variations in form of single nucleotide polymorphisms (SNPs). As very common alterations in NASH variants of the genes encoding patatin-like phospholipase domain-containing protein 2 (*PNPLA3*), transmembrane 6 superfamily member 2 (*TM6SF2*), membrane-bound O-acetyltransferase domain-containing protein 7 (*MBOAT7*) and glucokinase regulatory protein (*GCKR*) were identified. These genes are spread across the human genome (Figure 4).

The patatine-like phospholipase domain-containing protein 3 gene (*PNPLA3*) encodes for the triacylglycerol lipase adiponutrin that mediates the hydrolysis of triacylglycerol in adipocytes and hepatocytes. One of the most abundant DNA sequence variations associated with NAFLD and NASH is the isoleucine to methionine substitution in the *PNPLA3* gene at position 148 (PNPLA3-148M variant) [128,129]. This leads to a loss-of-function ending up in the accumulation of mutated 148M in hepatocytes and hepatic stellate cells where it further leads to the malignant storage of triglycerides [130]. Respective patients that are homozygous and carrying this variant have a tenfold increased risk to develop NAFLD-related HCC.

Transmembrane 6 superfamily member 2 (*TM6SF2*) is generally expressed in the liver and in the small intestine. It regulates the secretion of triglycerides and the content of lipid droplets as it is involved in VLDL (very low-density lipoprotein) secretion. The most frequent SNP is known in the *TM6SF2* gene is the E167K variant. This polymorphism is a loss-of-function mutation triggered by the replacement of glutamic acid by lysine at position 167. This mutation leads to the accumulation of triglycerides in hepatocytes and at the same time lowers the systemic lipoprotein levels [131].

A mutation leading to the replacement of cysteine by threonine is a common variant within the membrane bound O-acyltransferase domain-containing 7 (*MBOAT7*) gene. Recent investigations link this variant with a decrease in systemic and intrahepatic phosphatidyl-inositol containing arachidonic acid, thereby leading to an increased risk of getting NAFLD, NASH, and related end-stage liver diseases [132]. Another genetic variant, which was shown to be directly associated with the development of NAFLD by influencing the regulation of *de novo* lipogenesis and hepatic glucose uptake is the P446L mutation in the *GCKR* gene encoding for the glucokinase regulatory protein [133].

Besides those mentioned above, further genome-wide analysis show other gene variants associated with a higher risk of developing NAFLD and progress to related end-stage diseases. In this context, genetic polymorphisms of ethanol metabolizing enzymes (e.g., alcohol dehydrogenase) and cytochrome p450 2E1 (*CYP2E1*) activation triggering oxidative biotransformation and ROS formation, which is relevant in generating lipid peroxides, and their interference with the outcome of alcohol-induced liver disease and NASH has been discussed [134]. In particular, there are several clinical studies showing that alterations in *CYP2E1* activity are observed under various conditions, including obesity rendering respective persons more susceptible to liver injury [134,135]. Getting more knowledge about the exact effects influenced by those gene variations can be beneficial in the development of new therapeutic options and drug targets to treat NASH in the future.

More recently, several exploratory studies conducted in preclinical models identified circulating levels of non-coding RNA (ncRNA) such as microRNA and long ncRNA (lncRNA) to be associated with the pathogenesis and progression of various liver diseases [136]. In particular, several microRNAs representing a new class of highly conserved small non-coding RNA were shown to be critically involved in the regulation of complex gene networks in almost all acute and chronic liver disease [137]. As an example, profiling in diet-induced NASH progression and regression models identified the upregulation of a signature composed of six defined microRNA in NASH mice that allowed accurate distinguishing of NASH from lean mice [138]. However, in view of the large number of reported preclinical studies on miRNA, only a few have entered clinical trials and precise information about their diagnostic and prognostic value for human liver disease is still missing.

## 5. Screening for NAFLD and NASH

Identification of pre-symptomatic individuals or patients at risk would be the best to enable earlier disease intervention and management. There is a large number of early signs or symptoms indicating the onset of NAFLD, including central obesity, elevated serum triglyceride, and impaired fasting glucose. In addition, anorexia, nausea, vomiting, malaise, headache, or even epigastric and right upper quadrant abdominal pain, mild jaundice, and thrombocytopenia can already hint to initiation of NAFLD. Therefore, it was proposed that the potential of simple steatosis to progress into severe NAFLD necessitates timely detection of risk stratification in community-based healthcare settings [139]. However, despite multiple research reports demonstrating amazing promises, most of the proposed early protein or nucleic acid biomarkers are presently characterized by low sensitivity, low stability, and limited specificity [139]. In addition, screening programs, analyzing diagnostic panels are costly. Last but not least, NAFLD may be less benign than currently thought [33]. Therefore, there are limitations in defining diagnostic starting points for the management of prodomic phases of NAFLD. This is potentially the reason, why Scientific Societies such as the American Association for the Study of Liver Diseases (AASLD), the European Association for the Study of Liver Diseases (EASL), the National Institute for Health and Care Excellence (NICE), and the Asia-Pacific Working Party do not support a NAFLD screening program or only recommend screening programs in high-risk groups [140].

## 6. Other Factors in NAFLD and NASH

Most recently, first reports described that exosomes carrying a variety of cargoes, including proteins, fats, and various kinds of nucleic acids (mRNAs, microRNAs, other noncoding RNAs) have fundamental implications in liver pathobiology [141,142,143,144]. Although the precise mechanisms by which they contribute to NAFLD and NASH are still somewhat enigmatic, there are first reports proposing defined exosomal microRNAs such as miR-192 released from injured hepatocytes as potential new biomarkers to evaluate the progression from simple steatosis to NASH [145]. First encouraging studies have generated significant interest in exosomes as targets for biomarkers development [144].

Other researchers focus on potential roles of the adipose tissue in NAFLD. In particular, adipose tissue macrophages were proposed as key players in NAFLD [146]. There is a general consensus that a large set of signaling molecules such as lipids, microRNAs, adipokines and immune-related compounds are released from adipose tissue into the portal vein triggering hepatic inflammation [146]. In particular, the release of fatty acids from dysfunctional adipocytes results in liver parenchymal cell toxicity, which causes the ectopic accumulation of triglyceride-derived toxic metabolites increasing the activity of inflammatory pathways [147]. However, all these mechanisms are presently only partially understood and the impact of different macrophage phenotypes on the formation of NAFLD and NASH needs additional studies.

Another recent focus discussed in NAFLD and NASH research is the occurrence of quantitative and qualitative changes of the intestinal flora. Such a dysbiosis can result from altered food metabolism, intoxication, or increased permeability of the intestinal barrier. There is nowadays increasing evidence suggesting a critical role for the gut microbiome in the pathogenesis of obesity and metabolic syndrome [148]. The gut microbiota contributes to liver steatosis by modulating the uptake, bio-processing, fermentation, and synthesis of several effector molecules such as short-chain fatty acids, bile acids, cholines, and many other substances [149]. Moreover, it is intensively discussed at present if the microbiome composition can be used as a biomarker to differentiate between NAFLD and NASH [148]. However, standardized test systems or microbiota-targeted personalized treatment approaches for NAFLD and NASH are still not available.

## 7. Conclusions

Worldwide, NAFLD and NASH as well as NAFLD-related diseases (OSAS, PCOS, non-obese NAFLD), have emerged as leading causes of chronic liver disease in the last decades. The pathogenesis of respective diseases is complex and influenced by genetic factors, patients’ characteristics, and a variety of risk factors. Although the factors and involved pathways triggering initiating and progression of NAFLD and NASH are reasonably well known, there is an urgent clinical need to establish reliable, noninvasive biomarkers, tests or algorithms that avoid the need of liver biopsy and allow to differentiate between disease stages. During the last decades, a great variety of multiparametric panels and parameter combinations (NFLS, HIS, FLI, LAP index, FLIP, CHeK, NFS, APRI, BARD, ELF, Hepascore) taking into account serum markers (e.g., ALT, AST, bilirubin, Hb1Ac, HDL, α_2_M, platelet counts), patient characteristics (sex, gender, BMI), or comorbidities (diabetes) were established. However, all these diagnostic panels have limitations and alone are not suitable to replace liver biopsy. Nowadays, high-resolution imaging modalities such as ultrasound, MRI, elastography, and CAP have been established. Several of these techniques, such as MRI-PDFF, which can specifically detect the presence of hepatic steatosis and assess liver fat over the entire liver, might be useful to substitute biopsies and greatly assist the objective follow-up of therapeutic trials. Finally, liver-derived exosomes released into the systemic circulation and toxic lipids are in focus as targets for biomarkers development in liquid liver biopsies.

## Figures and Tables

**Table 1:** Blood biomarker panels to identify inflammatory liver disease.

Panel	Parameters Included	Reference
NAFLD ridge score	ALT, HDL, cholesterol, triglycerides, HbA1c, leukocyte count, hypertension	[43,44]
NAFLD Liver Fat Score (NLFS)	Liver fat content, metabolic syndrome, type 2 diabetes, AST, AST:ALT, fasting insulin level	[45]
Hepatic Steatosis Index (HIS)	AST:ALT, BMI, diabetes, sex	[46]
Fatty liver index (FLI)	BMI, waist circumference, triglycerides, γ-GT	[47,48]
Lipid accumulation product (LAP) index	sex, triglycerides, weight circumference	[49]
Fatty Liver Inhibition of Progression (FLIP) algorithm	histological steatosis, disease activity, fibrosis score	[50]
CHeK score	age, HbA1c, γ-GT, adiponectin, M30	[51]
NAFLD Fibrosis Score (NFS)	AST:ALT, albumin, platelet count, age, BMI, hyperglycemia (impaired fasting glucose)	[52]
Fibrosis-4 Score (FIB-4)	AST, ALT, platelet count, age	[53]
AST to Platelet Ratio Index (APRI)	AST:platelet count	[54]
BARD Score	BMI, AST:ALT, diabetes	[55]
Enhanced Liver Fibrosis panel (ELF)	Age, TIMP-1, PIIINP, HA	[56]
Hepascore	bilirubin, γ-GT, HA, α_2_M, age, gender	[57]
FibroTest-FibroSURE/ActiTest	α_2_M, haptoglobin, γ-GT, total bilirubin, apolipoprotein A1, ALT, age, gender	[58]
FibroMeter NAFLD index (FibroMeter^VCTE^)	platelet count, prothrombin index, ferritin, AST, ALT, body weight, age, liver stiffness determined by vibration controlled transient elastography (VCTE)	[59,60]

Abbreviations used are: α_2_M, α_2_-macroglobulin; γ-GT, γ-glutamyltransferase; ALT, alanine aminotransferase; AST, aspartate aminotransferase; BMI, body mass index; HA, hyaluronic acid; HbA1c, glycated hemoglobin; HDL, high-density lipoprotein; M30, antigen of the serum cytokeratin 18; PIIINP, amino-terminal propeptide of type III procollagen; TIMP-1, tissue inhibitor of metalloproteinases 1.
